# Supplementary material for: Nuclear localization of Argonaute 2 is affected by cell density and may relieve repression by microRNAs
Source: Nucleic Acids Res. 2023 Dec 18;52(4):1930–52. doi: 10.1093/nar/gkad1155 (PMC10899759; doi:10.1093/nar/gkad1155)
Supplement: gkad1155_Supplemental_File [file gkad1155_supplemental_file.pdf]

Supplemental Figures

Gene	Target	Primer Sequence 5'-3'
IGF1R	mRNA	F: TTCTGGACAAGCCAGACAAC<br>R: CTCCTCTTTGATGCTGCTGAT
IGF1R	Pre-mRNA	F: AGACAACTGTCCTGACATGC<br>R: TCCTAATCTCCTGTGACCCAA
OLFML2A	mRNA	F: CGACAGGATCTATGTCACCAAC<br>R: CTGTGCCGATCCAGTTGTAG
NOTCH3	mRNA	F: GCCAATAAGGACATGCAGGATAG<br>R: TGATCTCACGGTTGGCAAAG
RREB1	mRNA	F: GGCAGCTTGATAACACAAAGAAA<br>R: CCCTGCTGTAGCTATCATTCAC
RREB1	Pre-mRNA	F: AAGGAGGGCAGCTTGATAAC<br>R: TGACAATTAAACGGGAGGTAGG
RNF24	mRNA	F: CTTCAAGCCTCGAGATGAGTT<br>R: GGGACACACTTTACGAACCT
RNF24	Pre-mRNA	F: GAAGACTTCAAGCCTCGAGATG<br>R: CTCAAACTCGGGTCCCATTAAG
VEGFA	mRNA	F: CGAGTACATCTTCAAGCCATCC<br>R: GGTGAGGTTTGATCCGCATAAT
VEGFA	Pre-mRNA	F: GAGGGCAGAATCATCACGAA<br>R: AATTAGGCCATCCACCCATC
HPRT1	mRNA	F: AGTTCTGTGGCCATCTGCTTAGTAG<br>R: AAACAACAATCCGCCCAAAGG
snRNP200	mRNA	F: CGAGAAGTGGGACATCATCAC<br>R: ACCTCTGTCATCGTGGAGAA
ZMYM4	mRNA	F: GAGGGAAAGTGGAAGAGTTCTG<br>R: CTGAGTTTACCCTGTCGCTTAC
COL6A1	mRNA	F: CGCTGGTCAAGGAGAACTATG<br>R: CAGGTGTAATCTGGACACTTCTT
PRDM1	mRNA	F: GTATGCCACCAACAGTGAAGA<br>R: AGGAACTGTGTCATTGGTGTAG
MINK1	mRNA	F: GGCTCAAGTTCCTGTGTGAG<br>R: TCACCAGTTCATGATGCAGTTA
SERPINB9	mRNA	F: CTGGTGCTGCTGCCTGA<br>R: GGAGAACTTCAACCTCAGTACTCTTC

Supplemental Table 1. Sequences of primers used to test expression of candidate miRISC target genes and internal control genes.

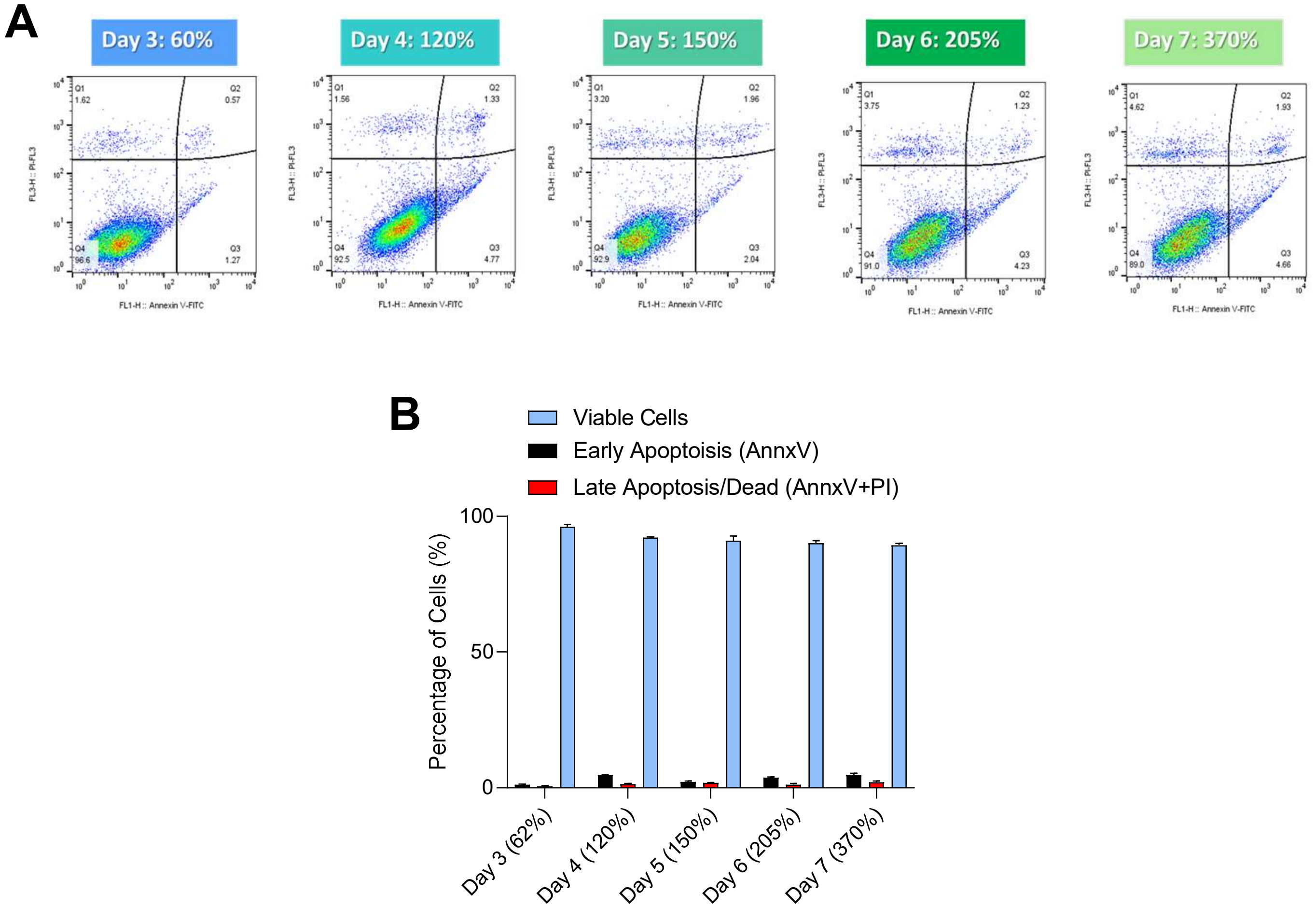

Supplemental Figure 1. Majority of HCT116 cells are viable throughout high cell density growth conditions.

(A) Scatter plots of flow cytometry data from cells grown to high cell density. Three biological replicates were performed for each cell density time point, and one representative plot is shown per day. (B) Bar graph summarizing dual-viability staining results in (A) with percentage of viable cells in blue, early apoptotic cells in black, and late apoptotic/dead cells in red. Statistical significance calculated using multiple paired t-tests relative to Day 3. HCT116 cells stained with Annexin V-FITC (AnnxV) and propidium iodide (PI) after growing for a total of seven days with Day 3-7 shown.

A

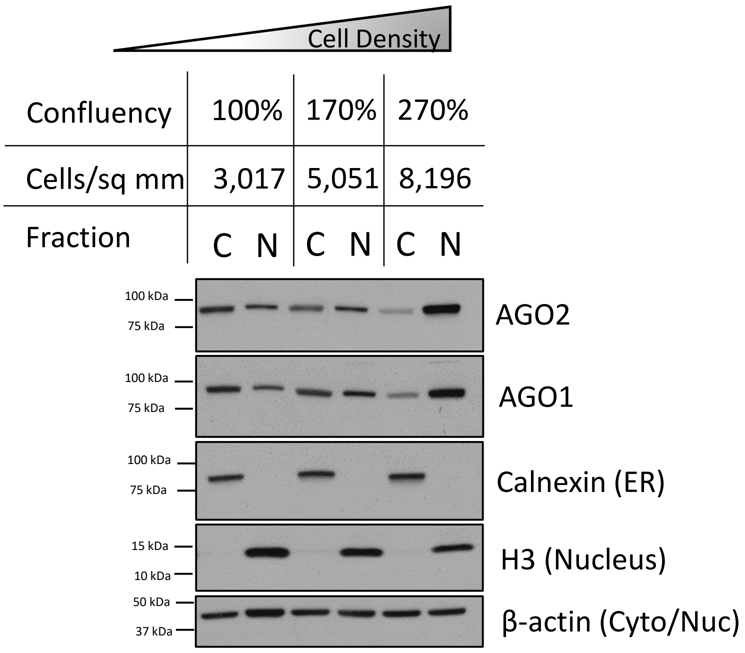

B

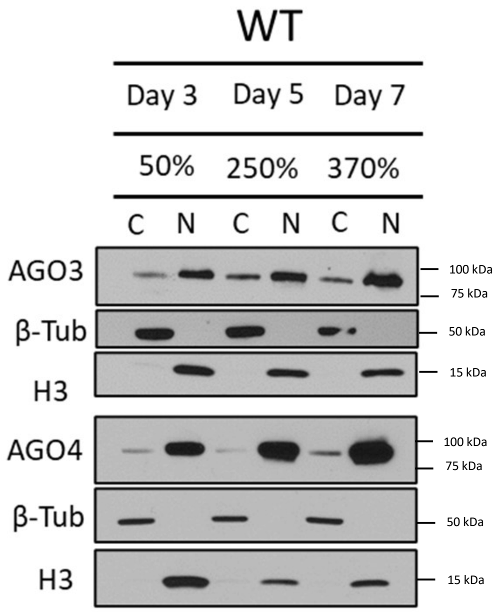

Supplemental Figure 2. AGO1-4 localization as a function of cell density.
(A) Representative Western blot showing AGO1 and AGO2 localization as a function of cell density. Same cell lysate samples as in Figure 1D with AGO1 localization shown. (B) Representative Western blot showing AGO3 and AGO4 localization as a function of cell density.

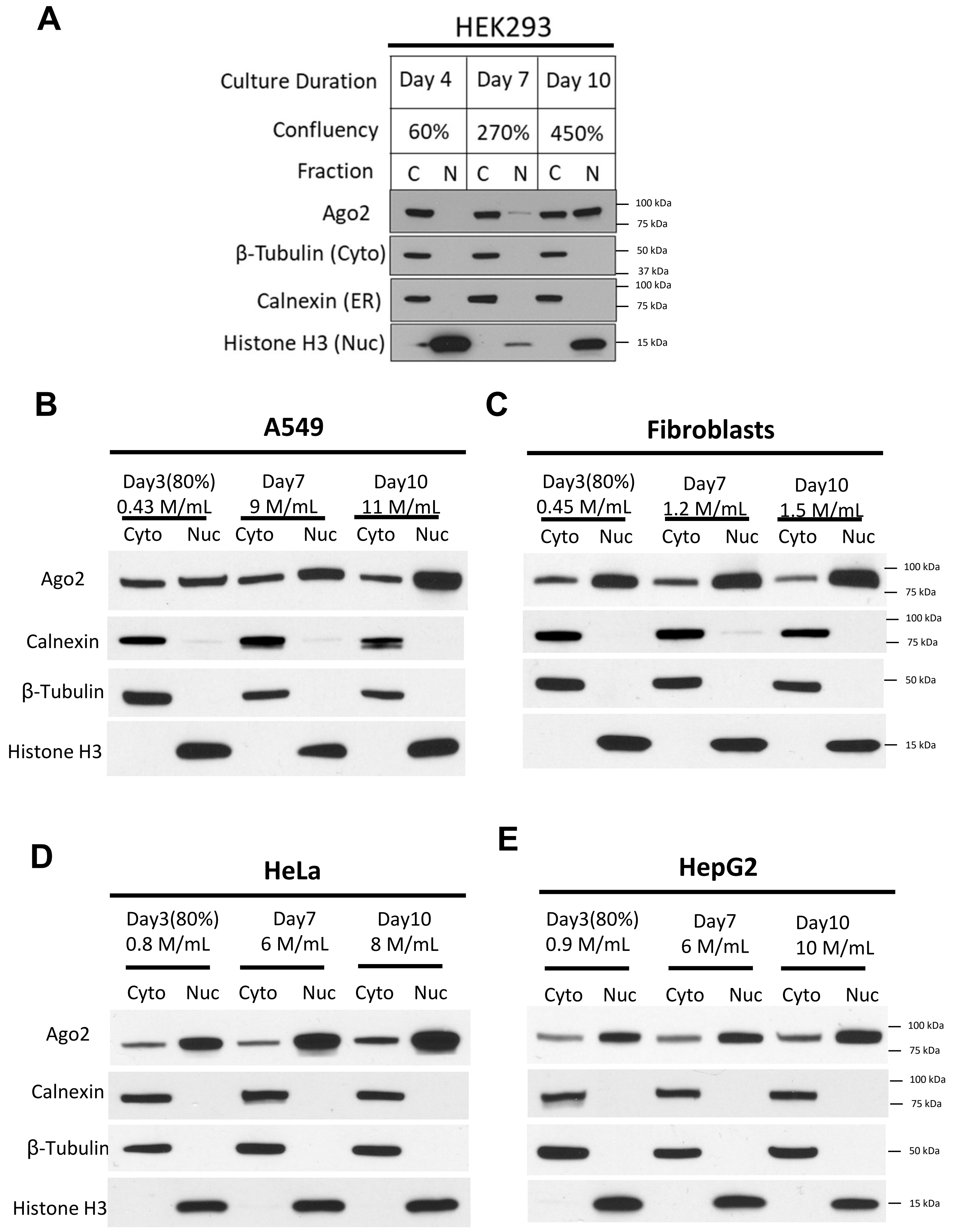

Supplemental Figure 3. AGO2 localization in other cell lines. Western blot showing AGO2 localization of different cell lines grown to high cell density. (A) HEK293, (B) A549, (C) fibroblasts, which do not grow beyond a monolayer, (D) HeLa, and (E) HepG2.

A

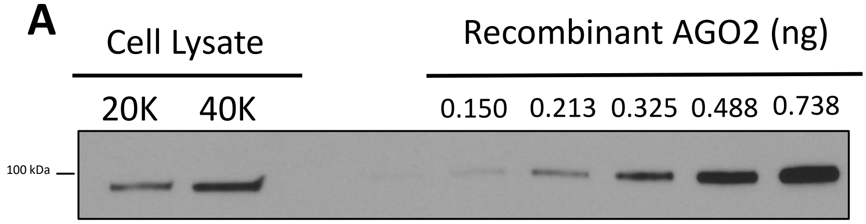

Lane	Signal	Mass Calculation	Cells	AGO2 Molecules	AGO2 Copy per cell
1	12060.539	0.274464815	20,000	1.65E+09	82,614
2	24734.731	0.471869837	40,000	2.84E+09	71,016
				**Average**	**76,815**

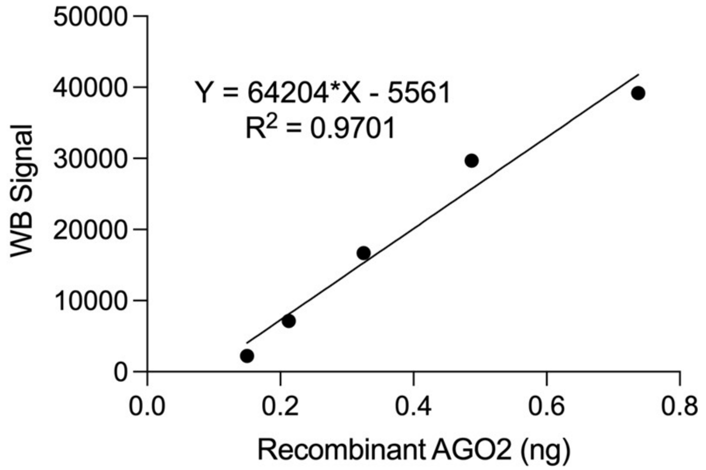

B

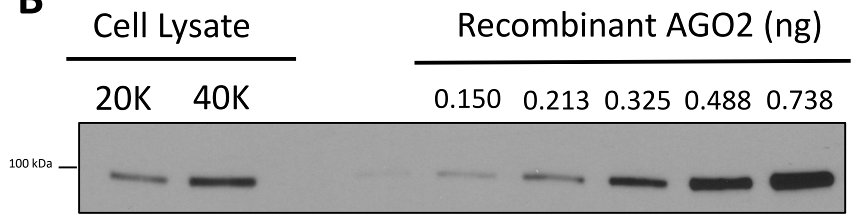

Lane	Signal	Mass Calculation	Cells	AGO2 Molecules	AGO2 Copy per cell
1	8683.468	0.199837	20,000	1.20E+09	60,151
2	21690.075	0.392359	40,000	2.36E+09	59,050
				**Average**	**59,600**

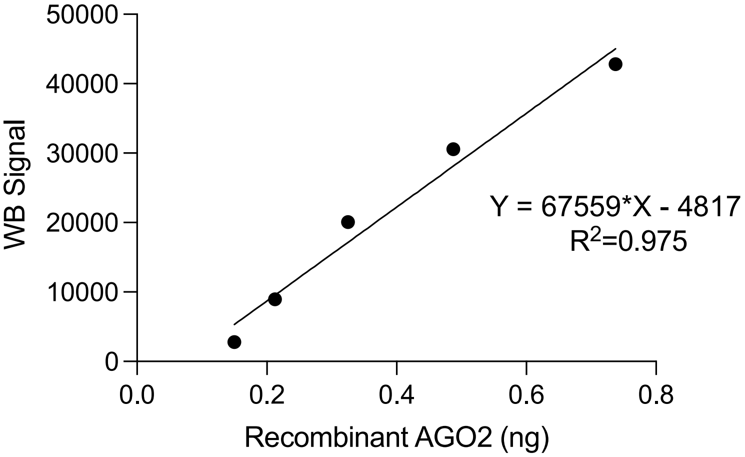

C

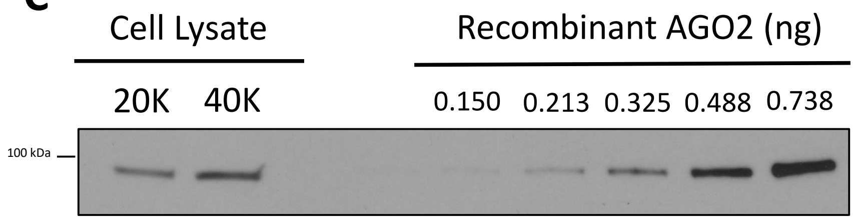

Lane	Signal	Mass Calculation	Cells	AGO2 Molecules	AGO2 Copy per cell
1	12203.711	0.298088	20,000	1.79E+09	89,724
2	22266.368	0.427861	40,000	2.58E+09	64,393
				**Average**	**77,059**

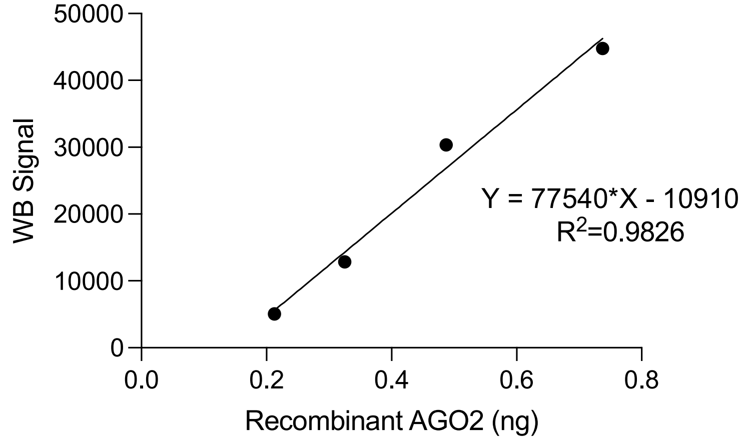

Supplementary Figure 4. (A-C) Western blots, linear regressions and calculated mass and copy number of AGO2 for Day 3 whole cell absolute quantification. Densitometry was used to produce linear regressions from a serial dilution of recombinant AGO2. Mass of AGO2 per lysate sample was calculated based on linear regression and AGO2 molecular weight and number of cells loaded per lysate were used to calculate number of AGO2 molecules and copy number per cell.

D

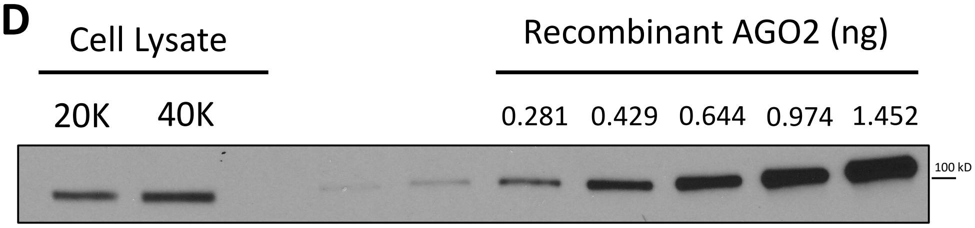

Lane	Signal	Mass Calculation	Cells	AGO2 Molecules	AGO2 Copy per cell
1	14351.832	0.26888	20,000	1.62E+09	80,933
2	21457.075	0.475403	40,000	2.86E+09	71,548
				**Average**	**76,240**

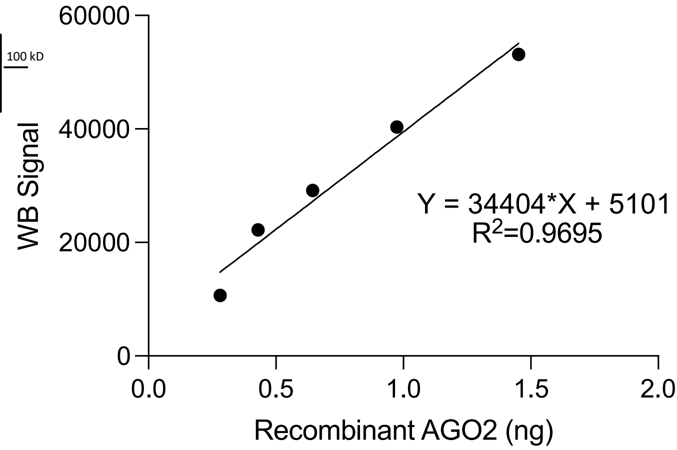

E

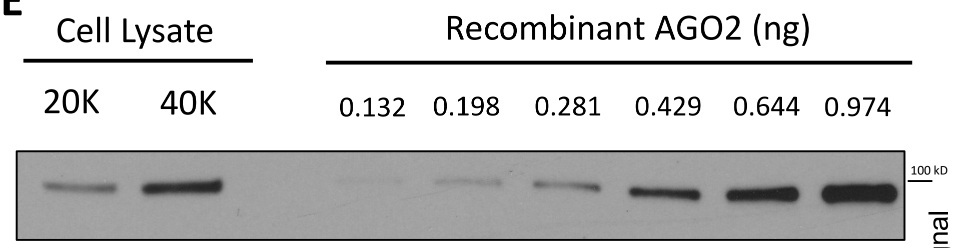

Lane	Signal	Mass Calculation	Cells	AGO2 Molecules	AGO2 Copy per cell
1	8457.175	0.257588	20,000	1.55E+09	77,534
2	25794.61	0.595846	40,000	3.59E+09	89,675
				**Average**	**83,604**

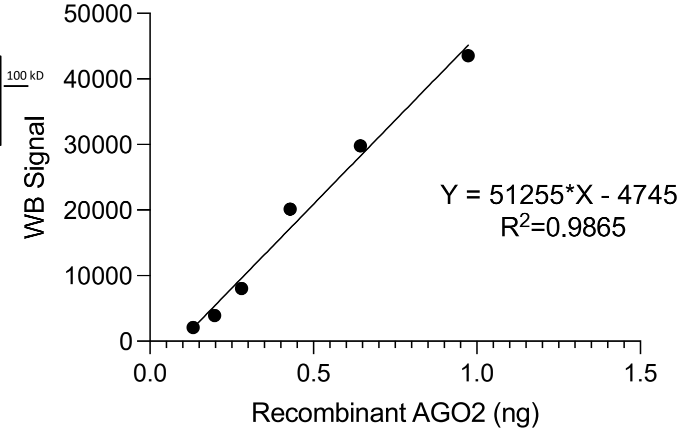

F

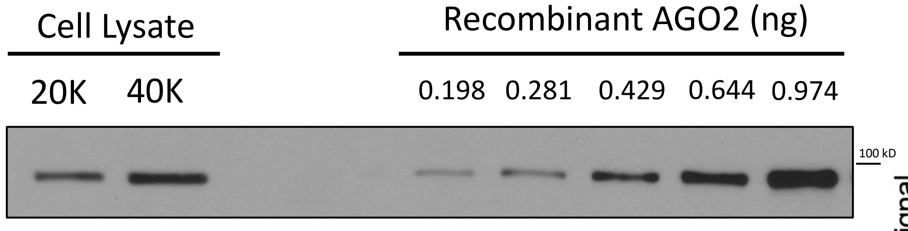

Lane	Signal	Mass Calculation	Cells	AGO2 Molecules	AGO2 Copy per cell
1	13455.711	0.373897	20,000	2.25E+09	112,543
2	26633.024	0.641039	40,000	3.86E+09	96,476
				**Average**	**104,510**

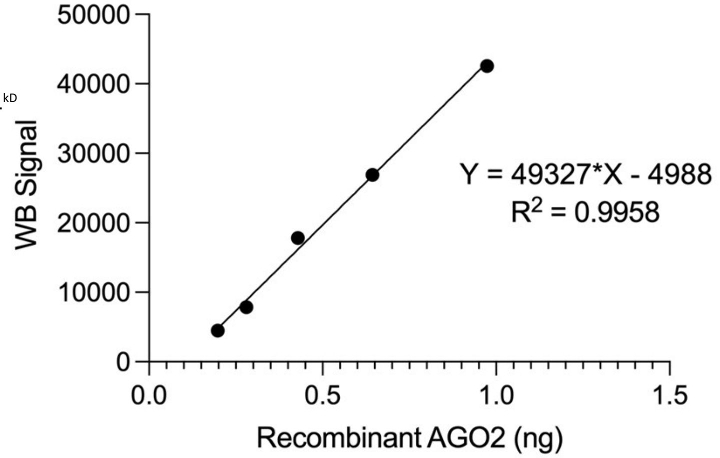

Supplementary Figure 4. (D-F) Western blots, linear regressions and calculated mass and copy number of AGO2 for Day 7 whole cell absolute quantification. Densitometry was used to produce linear regressions from a serial dilution of recombinant AGO2. Mass of AGO2 per lysate sample was calculated based on linear regression and AGO2 molecular weight and number of cells loaded per lysate were used to calculate number of AGO2 molecules and copy number per cell.

G

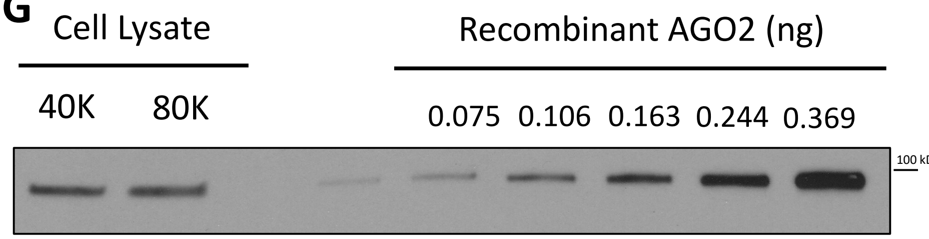

Lane	Signal	Mass Calculation	Cells	AGO2 Molecules	AGO2 Copy per cell
1	23387.874	0.171201	40,000	1.03E+09	25,766
2	24344.267	0.177283	80,000	1.07E+09	13,341
				**Average**	**19,553**

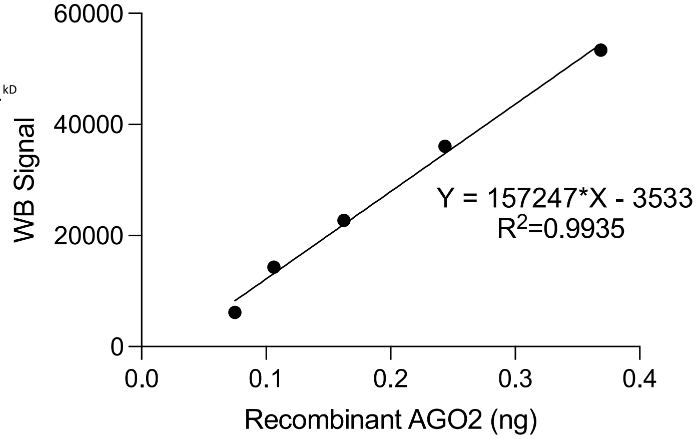

H

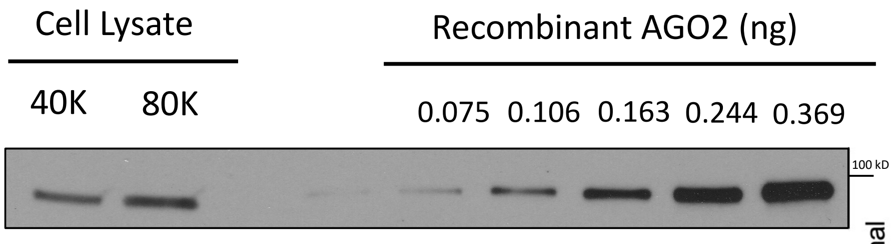

Lane	Signal	Mass Calculation	Cells	AGO2 Molecules	AGO2 Copy per cell
1	16558.61	0.119355	40,000	7.19E+08	17,963
2	26173.388	0.161011	80,000	9.69E+08	12,116
				**Average**	**15,039**

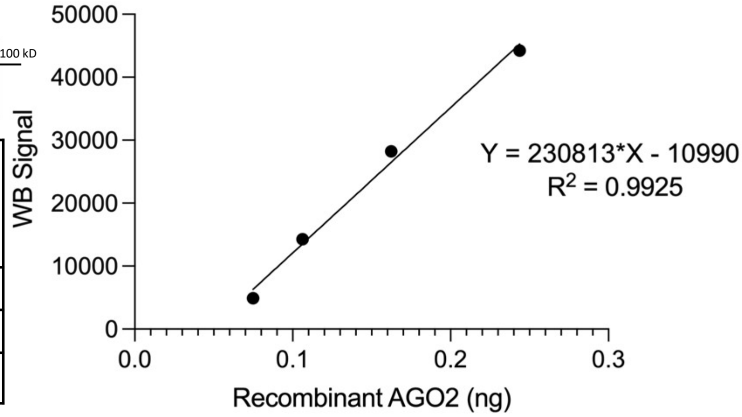

I

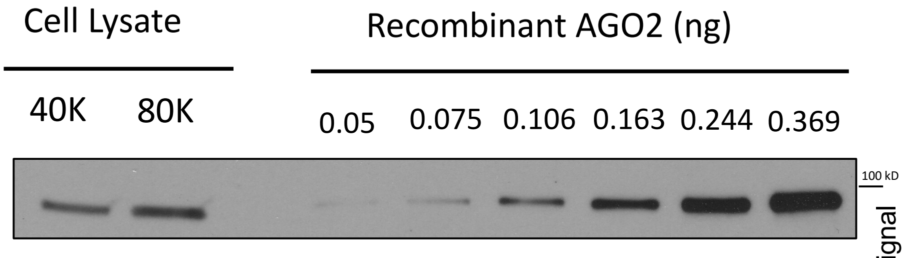

Lane	Signal	Mass Calculation	Cells	AGO2 Molecules	AGO2 Copy per cell
1	14299.075	0.091322	40,000	5.50E+08	13,744
2	16598.267	0.101599	80,000	6.12E+08	7,645
				**Average**	**10,695**

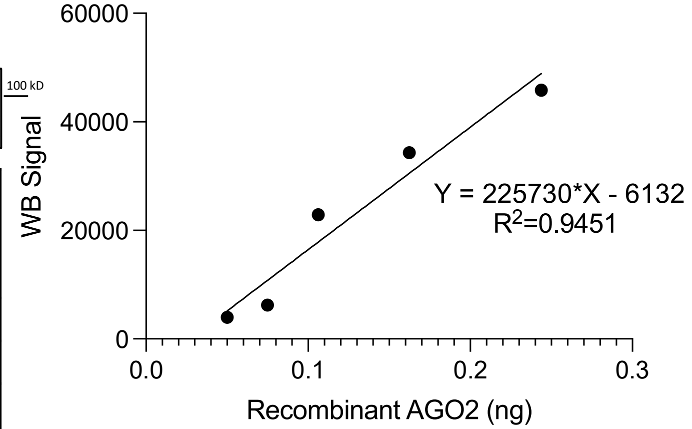

Supplementary Figure 4. (G-I) Western blots, linear regressions and calculated mass and copy number of AGO2 for Day 3 cytoplasmic fraction absolute quantification. Densitometry was used to produce linear regressions from a serial dilution of recombinant AGO2. Mass of AGO2 per lysate sample was calculated based on linear regression and AGO2 molecular weight and number of cells loaded per lysate were used to calculate number of AGO2 molecules and copy number per cell.

J

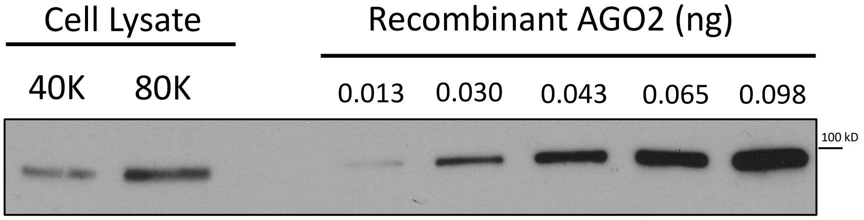

Lane	Signal	Mass Calculation	Cells	AGO2 Molecules	AGO2 Copy per cell
1	10477.024	0.021886	40,000	1.32E+08	3,294
2	24615.288	0.049577	80,000	2.98E+08	3,731
				**Average**	**3,512**

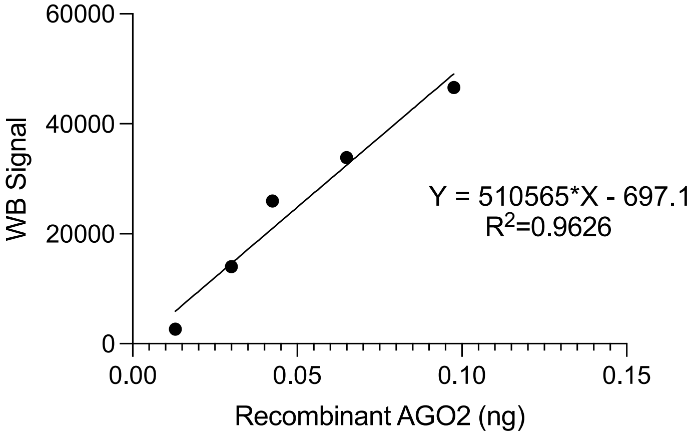

K

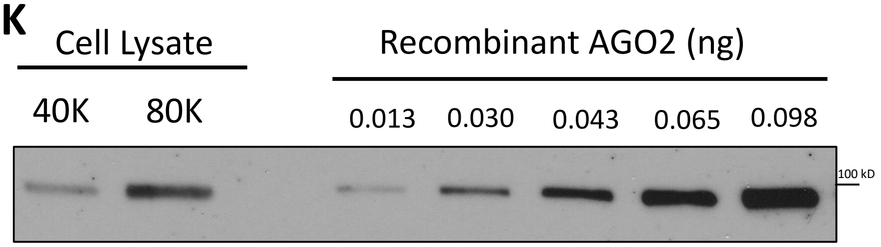

Lane	Signal	Mass Calculation	Cells	AGO2 Molecules	AGO2 Copy per cell
1	7529.903	0.018709	40,000	1.13E+08	2,816
2	24938.095	0.052412	80,000	3.16E+08	3,944
				**Average**	**3,380**

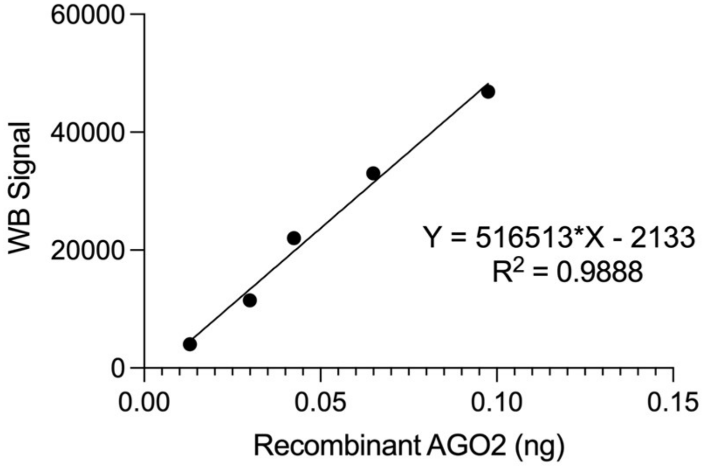

L

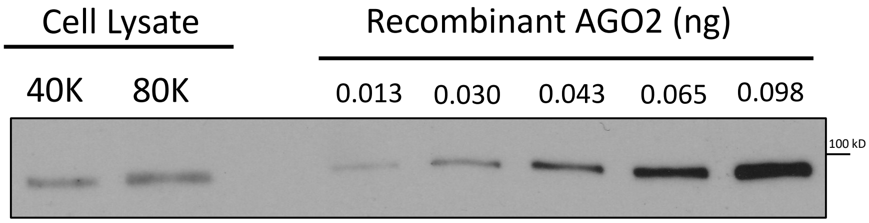

Lane	Signal	Mass Calculation	Cells	AGO2 Molecules	AGO2 Copy per cell
1	8439.418	0.026955	40,000	1.62E+08	4,057
2	15942.631	0.040485	80,000	2.44E+08	3,046
				Average	3,552

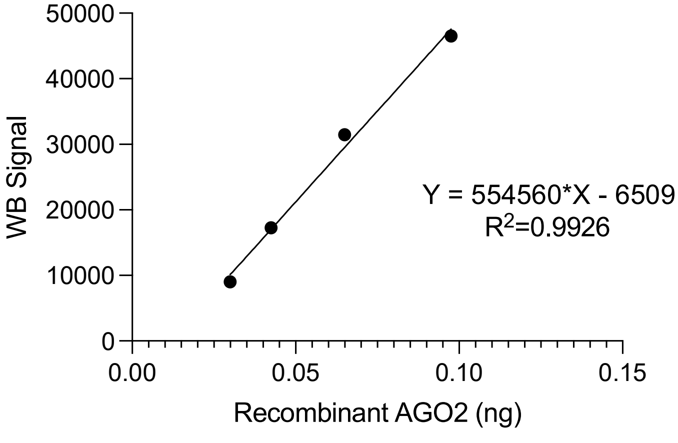

Supplementary Figure 4. (J-L) Western blots, linear regressions and calculated mass and copy number of AGO2 for Day 7 cytoplasmic fraction absolute quantification. Densitometry was used to produce linear regressions from a serial dilution of recombinant AGO2. Mass of AGO2 per lysate sample was calculated based on linear regression and AGO2 molecular weight and number of cells loaded per lysate were used to calculate number of AGO2 molecules and copy number per cell.

M

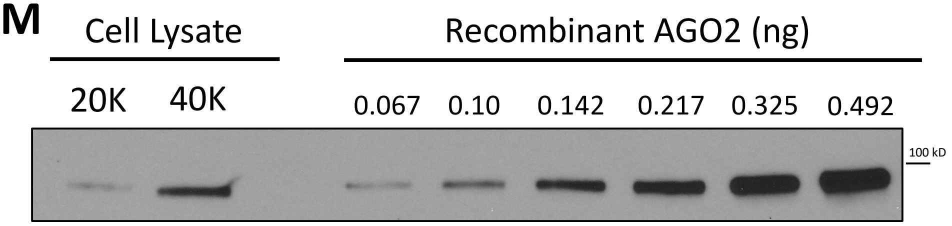

Lane	Signal	Mass Calculation	Cells	AGO2 Molecules	AGO2 Copy per cell
2	16213.146	0.171872	40,000	1.03E+09	25,867
				**Average**	**25,867**

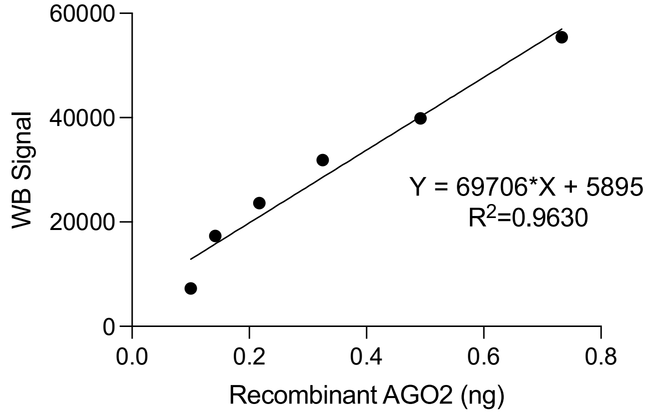

N

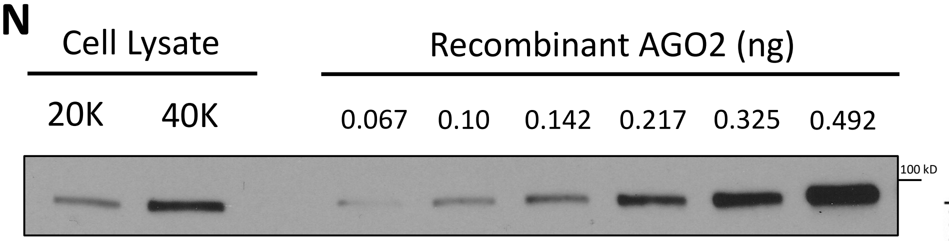

Lane	Signal	Mass Calculation	Cells	AGO2 Molecules	AGO2 Copy per cell
1	6572.882	0.102296	20,000	6.16E+08	30,791
2	21230.075	0.256535	40,000	1.54E+09	38,609
				**Average**	**34,700**

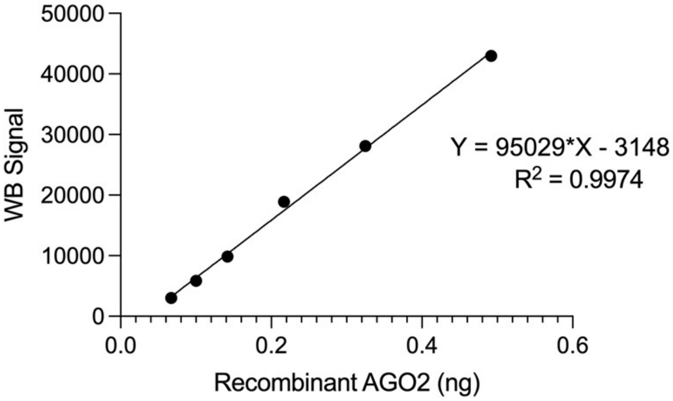

O

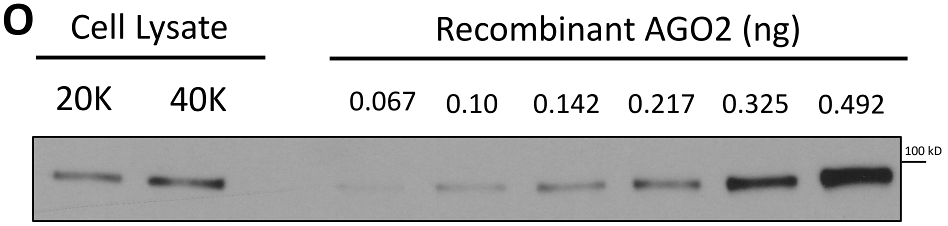

Lane	Signal	Mass Calculation	Cells	AGO2 Molecules	AGO2 Copy per cell
1	7910.711	0.16049	20,000	9.66E+08	48,308
2	15390.539	0.24147	40,000	1.45E+09	36,341
				**Average**	**42,324**

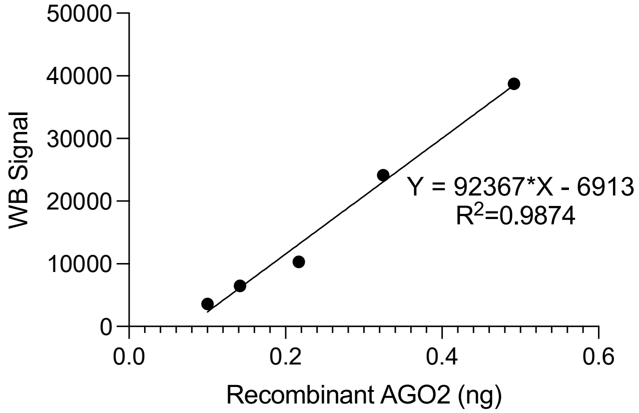

Supplementary Figure 4. (M-O) Western blots, linear regressions and calculated mass and copy number of AGO2 for day 3 nuclear fraction absolute quantification. Densitometry was used to produce linear regressions from a serial dilution of recombinant AGO2. Mass of AGO2 per lysate sample was calculated based on linear regression and AGO2 molecular weight and number of cells loaded per lysate were used to calculate number of AGO2 molecules and copy number per cell.

P

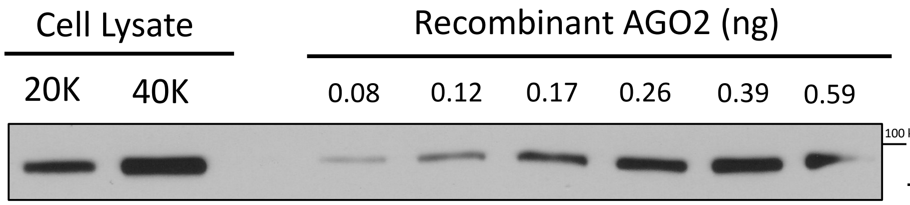

Lane	Signal	Mass Calculation	Cells	AGO2 Molecules	AGO2 Copy per cell
1	40199.338	0.231832	20,000	1.40E+09	69,781
2	74011.016	0.386894	40,000	2.33E+09	58,228
				**Average**	**64,004**

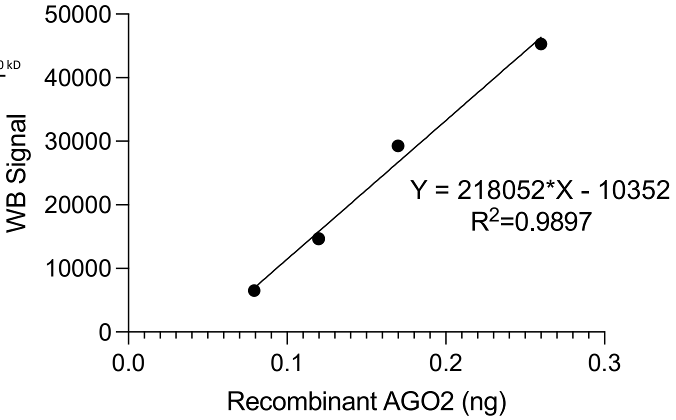

Q

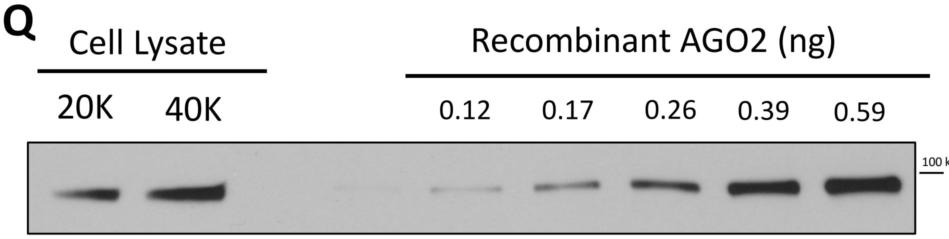

Lane	Signal	Mass Calculation	Cells	AGO2 Molecules	AGO2 Copy per cell
1	18464.418	0.260777	20,000	1.57E+09	78,494
2	34665.439	0.436065	40,000	2.63E+09	65,628
				**Average**	**72,061**

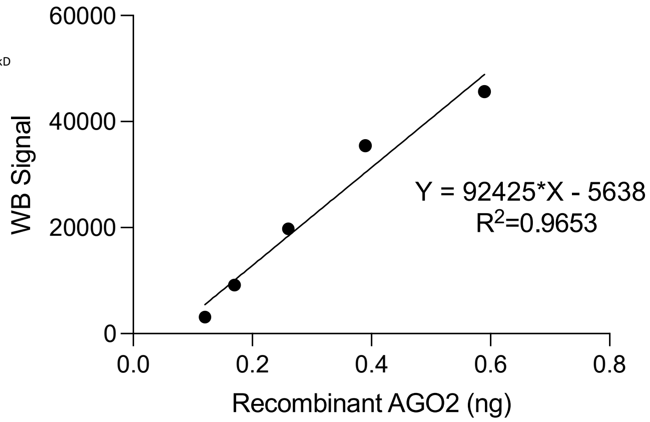

R

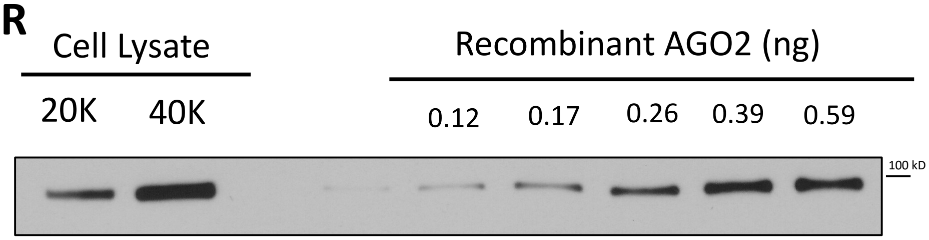

Lane	Signal	Mass Calculation	Cells	AGO2 Molecules	AGO2 Copy per cell
1	26987.731	0.277917	20,000	1.67E+09	83,653
2	63852.167	0.550536	40,000	3.31E+09	82,856
				**Average**	**83,254**

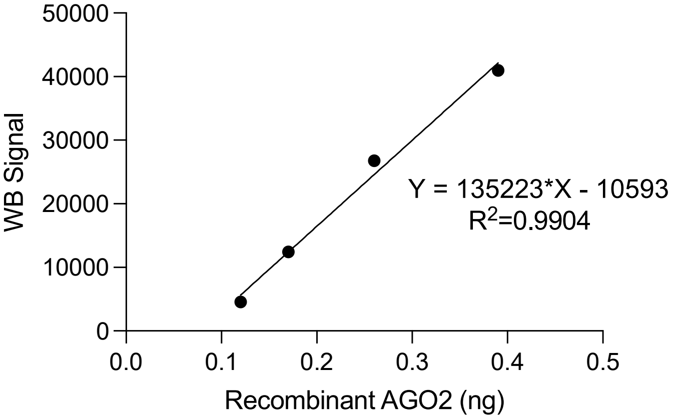

Supplementary Figure X. (P-R) Western blots, linear regressions and calculated mass and copy number of AGO2 for day 7 nuclear fraction absolute quantification. Densitometry was used to produce linear regressions from a serial dilution of recombinant AGO2. Mass of AGO2 per lysate sample was calculated based on linear regression and AGO2 molecular weight and number of cells loaded per lysate were used to calculate number of AGO2 molecules and copy number per cell.

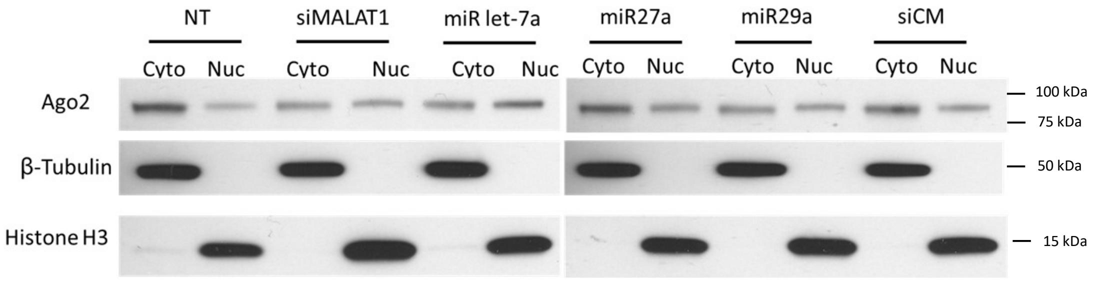

Supplementary Figure 5. AGO2 nuclear localization is sensitive to microRNA depletion. (A) Western blot showing transfection of microRNA mimics and control oligos into Drosha-/- cells rescues nuclear AGO2 localization.

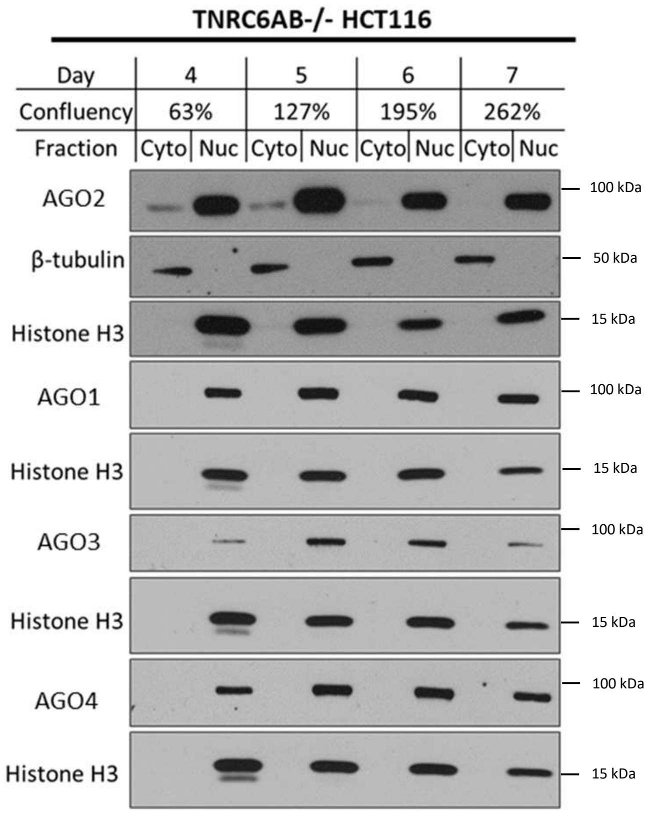

Supplementary Figure 6. Western blots as in Figure 2C. AGO1-4 were run on separate gels with individual Histone H3 shown as loading control and nuclear purity marker for each gel.

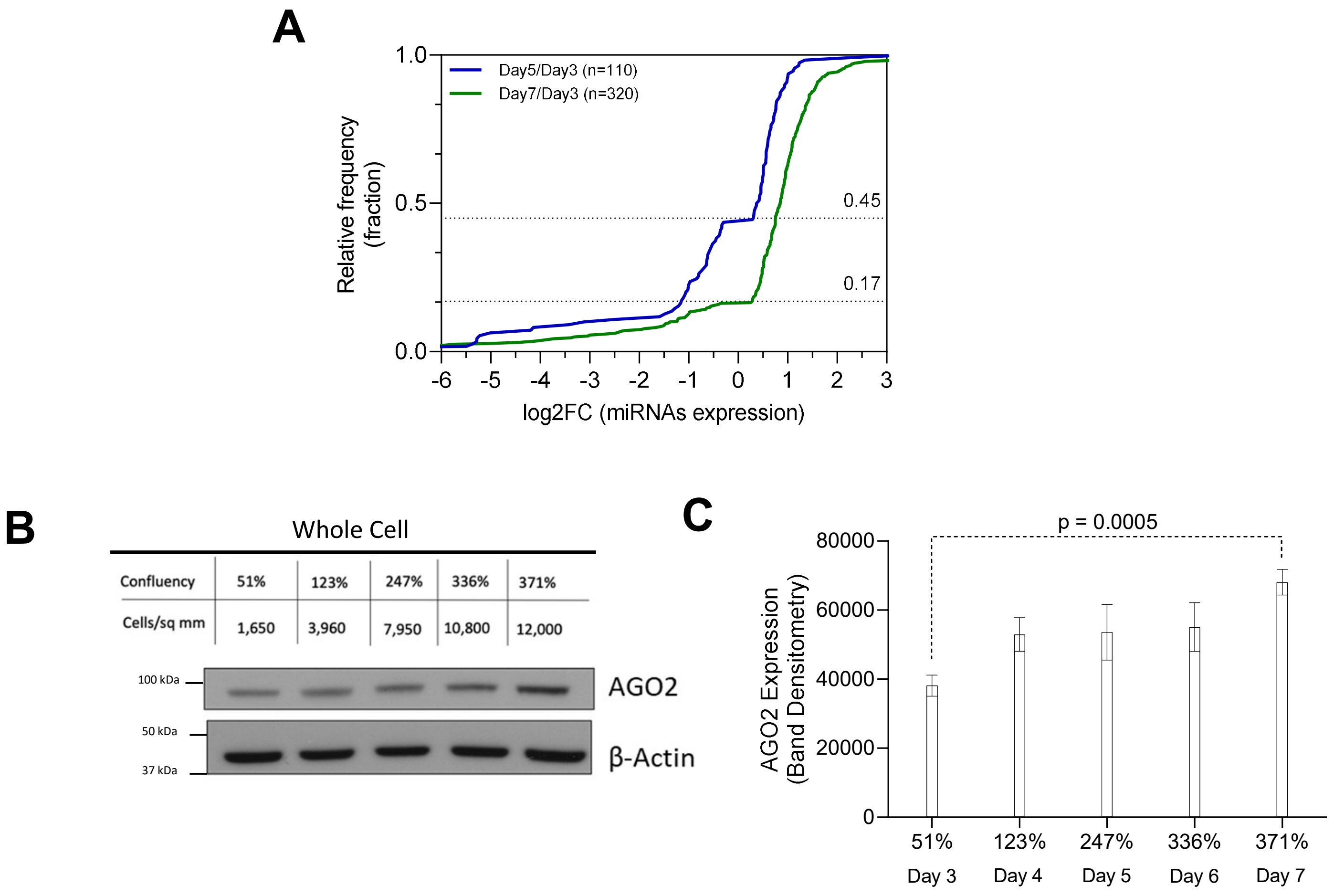

Supplemental Figure 7. Global miRNA expression increase matches increase in total AGO2 protein levels. (A) Cumulative distribution frequency plot showing that over 80% of significantly effected miRNAs by Day 7 are upregulated. (B) Western blot showing AGO2 expression in whole cell HCT116 at increasing cell density. (C) Quantification of AGO2 band densitometry relative to β-Actin in (B). Increase in AGO2 is significant on Day 7 relative to Day 3. Unpaired t-test with Welch's correction to calculate p-value of 0.0005.

A

		Cyto-3'UTR-No6mer			
miRNA Rank by Abundance	Total miRNA Family Seeds: 8	Total AGO2 Binding Sites: 29	Seed	log2FC(Day7/Day3)	padj
#1	miR-10	IGF1R	7mer-a1	2.07	2.9E-124
		IGF1R	7mer-m8		
		IGF1R	7mer-a1		
		IGF1R	8mer		
		IGF1R	7mer-a1		
		IGF1R	8mer		
		IGF1R	8mer		
#3	let-7a	IGF1R	7mer-a1		
		IGF1R	7mer-a1		
		IGF1R	7mer-a1		
		IGF1R	7mer-a1		
		IGF1R	7mer-a1		
		IGF1R	7mer-a1		
		IGF1R	7mer-a1		
		IGF1R	7mer-a1		
		IGF1R	7mer-a1		
#8	miR-22	IGF1R	7mer-a1		
#9	miR-27	IGF1R	7mer-a1		
#10	miR-182	IGF1R	7mer-a1		
#13	miR-15	IGF1R	7mer-m8		
		IGF1R	7mer-m8		
		IGF1R	7mer-m8		
		IGF1R	7mer-m8		
		IGF1R	7mer-m8		
		IGF1R	7mer-m8		
		IGF1R	7mer-m8		
		IGF1R	7mer-m8		
#16	miR-17	IGF1R	7mer-a1		
#17	miR-181	IGF1R	8mer		

B

		Cyto-3'UTR-No6mer			
miRNA Rank by Abundance	Total miRNA Family Seeds: 1	Total AGO2 Binding Sites: 2	Seed	log2FC(Day7/Day3)	padj
#9	miR-27	NOTCH3	7mer-a1	3.88	3.5E-73
		NOTCH3	7mer-m8		

C

		Cyto-3'UTR-No6mer			
miRNA Rank by Abundance	Total miRNA Family Seeds: 2	Total AGO2 Binding Sites: 2	Seed	log2FC(Day7/Day3)	padj
#10	miR-182	OLFML2A	7mer-a1	2.60	4.6E-81
#12	miR-29	OLFML2A	7mer-m8		

Supplemental Figure 8. miRNAs with seed matches within regions of AGO2 binding sites for top three candidate miRISC targets. (A-C) microRNAs that have binding sites within regions of AGO2 occupancy from AGO2-eCLIP-sequencing within (A) IGF1R, (B) NOTCH3, and (C) OLFML2A 3’UTR in cytoplasm.

A

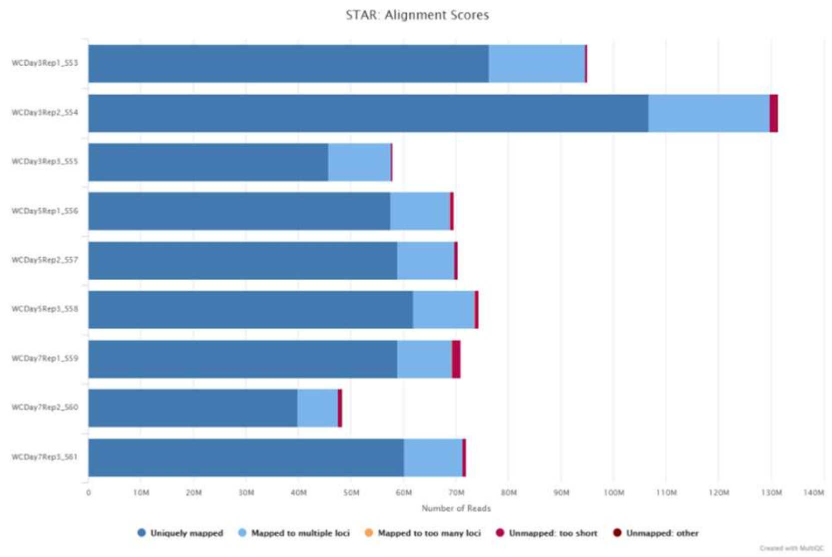

B

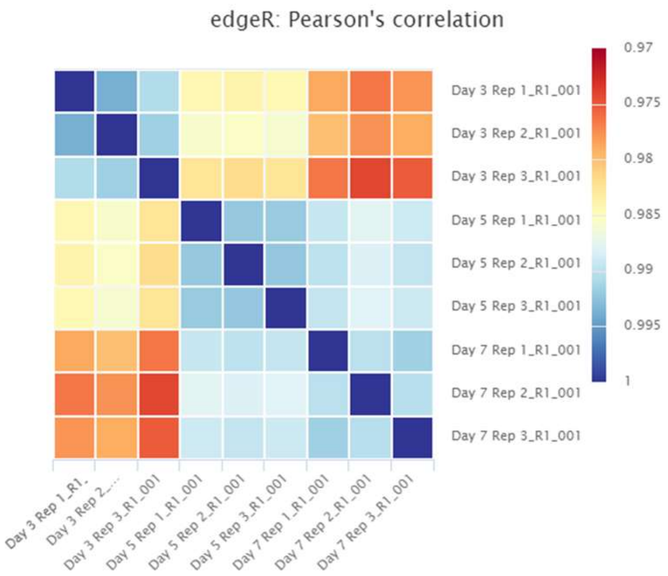

C

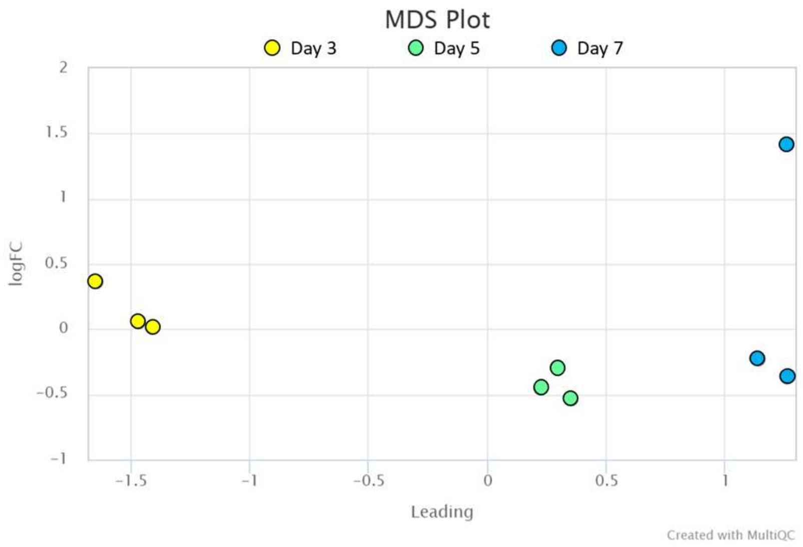

Supplemental Figure 9. Whole Transcriptome Sequencing Quality Control. (A) STAR Alignment Scores describing unique reads. (B) edgeR: Sample Similarity is generated from normalized gene counts through edgeR. Pearson's correlation between log2 normalized CPM values are then calculated and clustered. (C) Multidimensional scaling (MDS) plot showing clustering of each replicate submitted for whole transcriptome sequencing. (A-C) Plots generated by MultiQC.

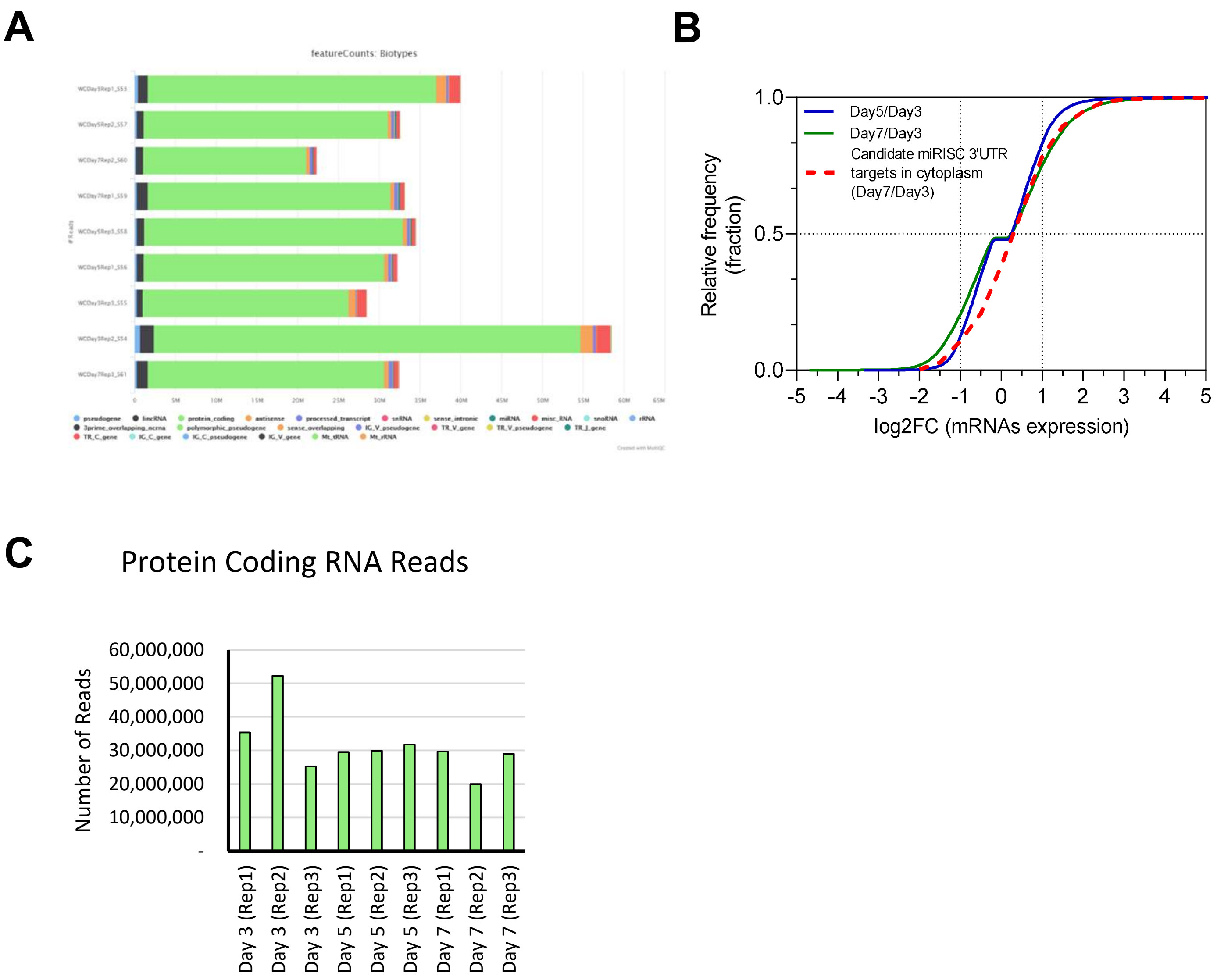

Supplemental Figure 10. (A) Biotype Counts, from whole transcriptome RNA-sequencing analysis, show reads overlapping genomic features of different biotypes, counted by featureCounts**.** (B) Cumulative distribution frequency plot showing global differential expression on Day 5 relative to Day 3 (blue), Day 7 relative to Day 3 (green), and the subset of candidate cytoplasm miRISC targets (dotted red line, all genes from Fig 5B). (C) Biotype counts from (A) showing reads aligning to protein coding RNAs.

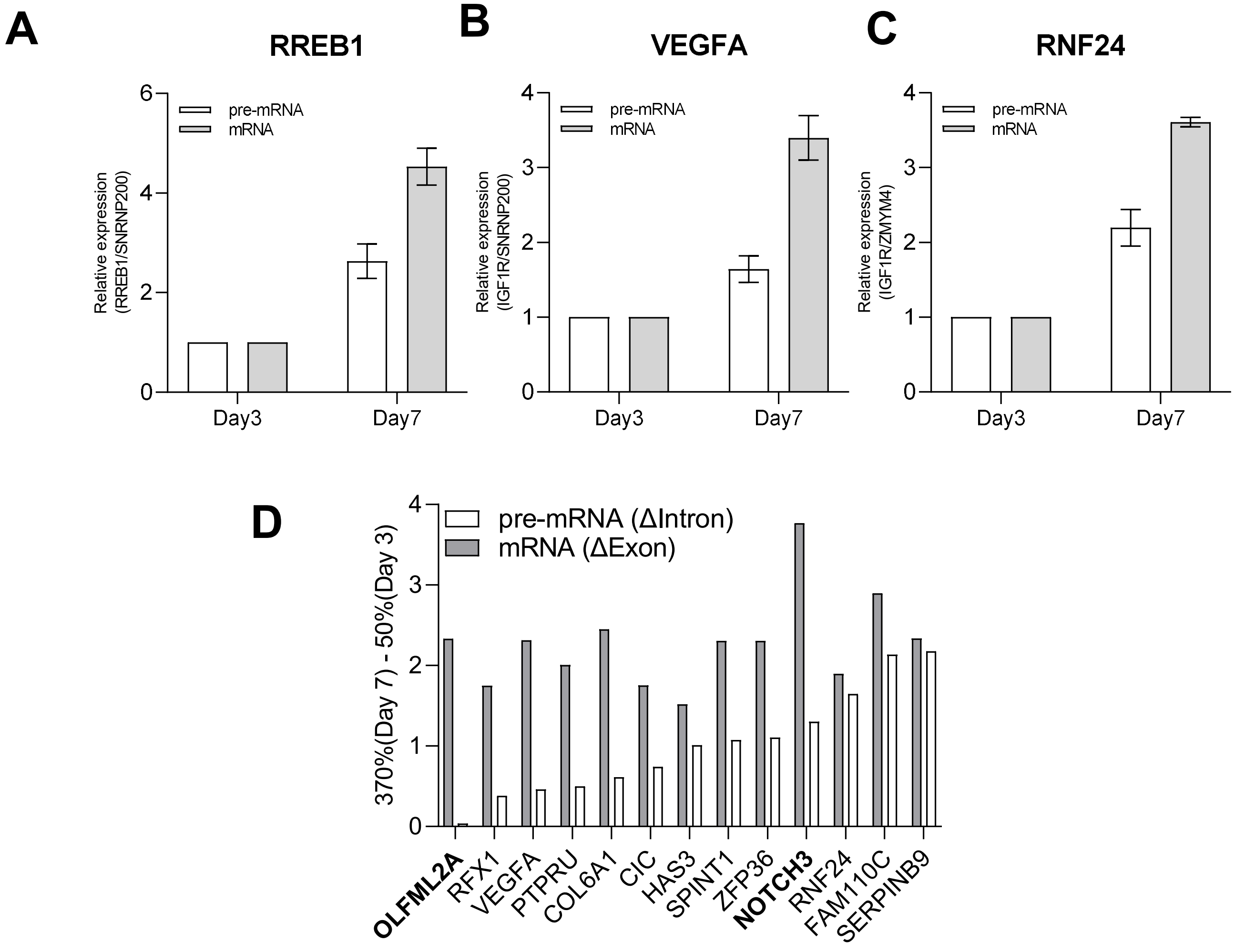

Supplemental Figure 11. (A-C) RT-qPCR measurements of relative expression of pre-mRNA and mRNA for candidate cytoplasmic RISC targets on Day 7 (370% confluent) relative to Day 3 (50% confluent). (D) Exon Intron Split Analysis results measuring relative changes in pre-mRNA (ΔIntron) and mRNA (ΔExon) levels from RNA-sequencing.

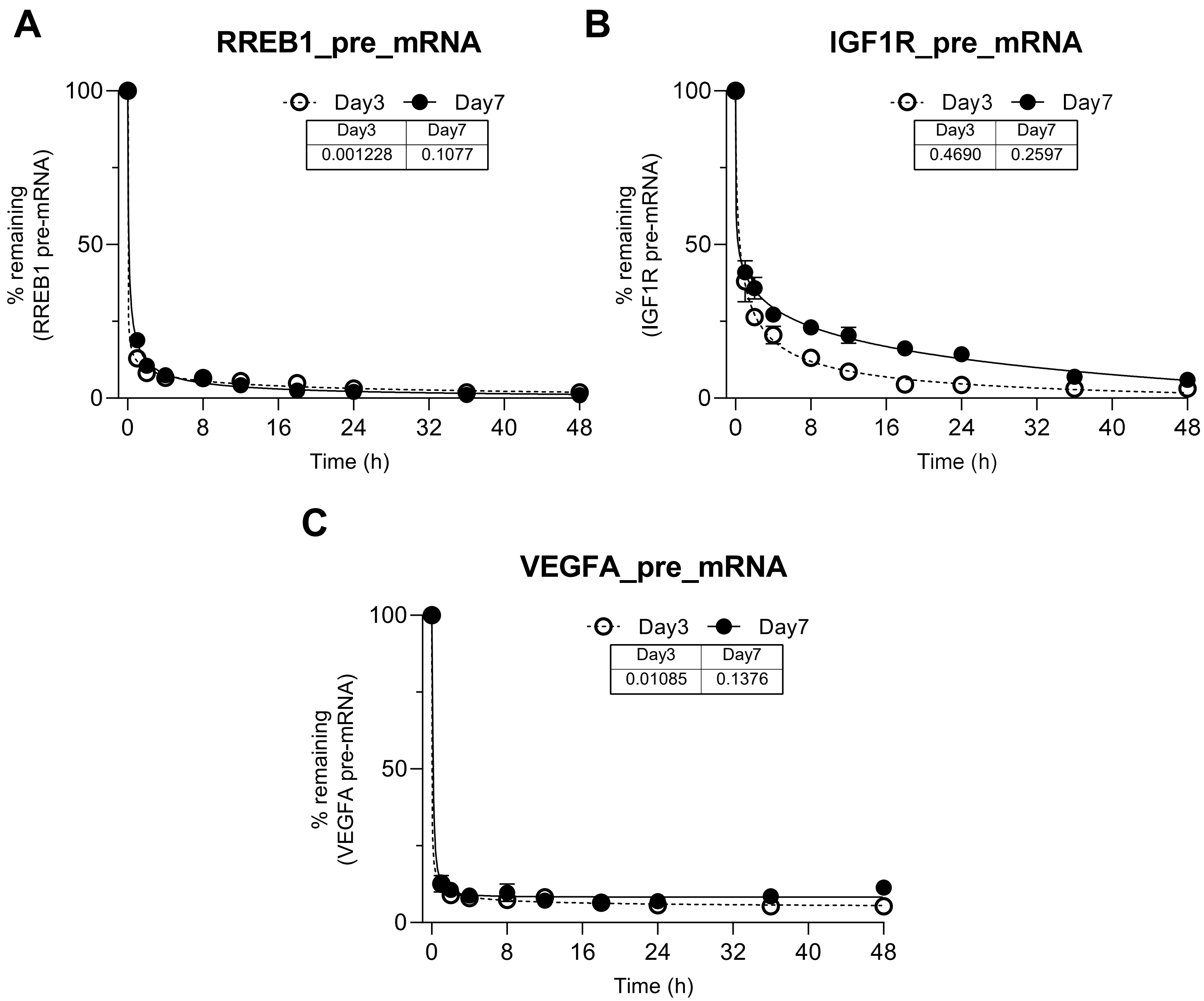

Supplemental Figure 12. (A-C) pre-mRNA stability assays for upregulated targets in RNA isolated from nuclear fraction from cells harvested on Day 3 (~50%) and Day 7 (~370%). RT-qPCR validating Actinomycin D treatment shut-down transcription at similar time after treatment in cells grown for 3 and 7 days. Transcription shut-down occurs within first hour independent of cell confluency (N=3).

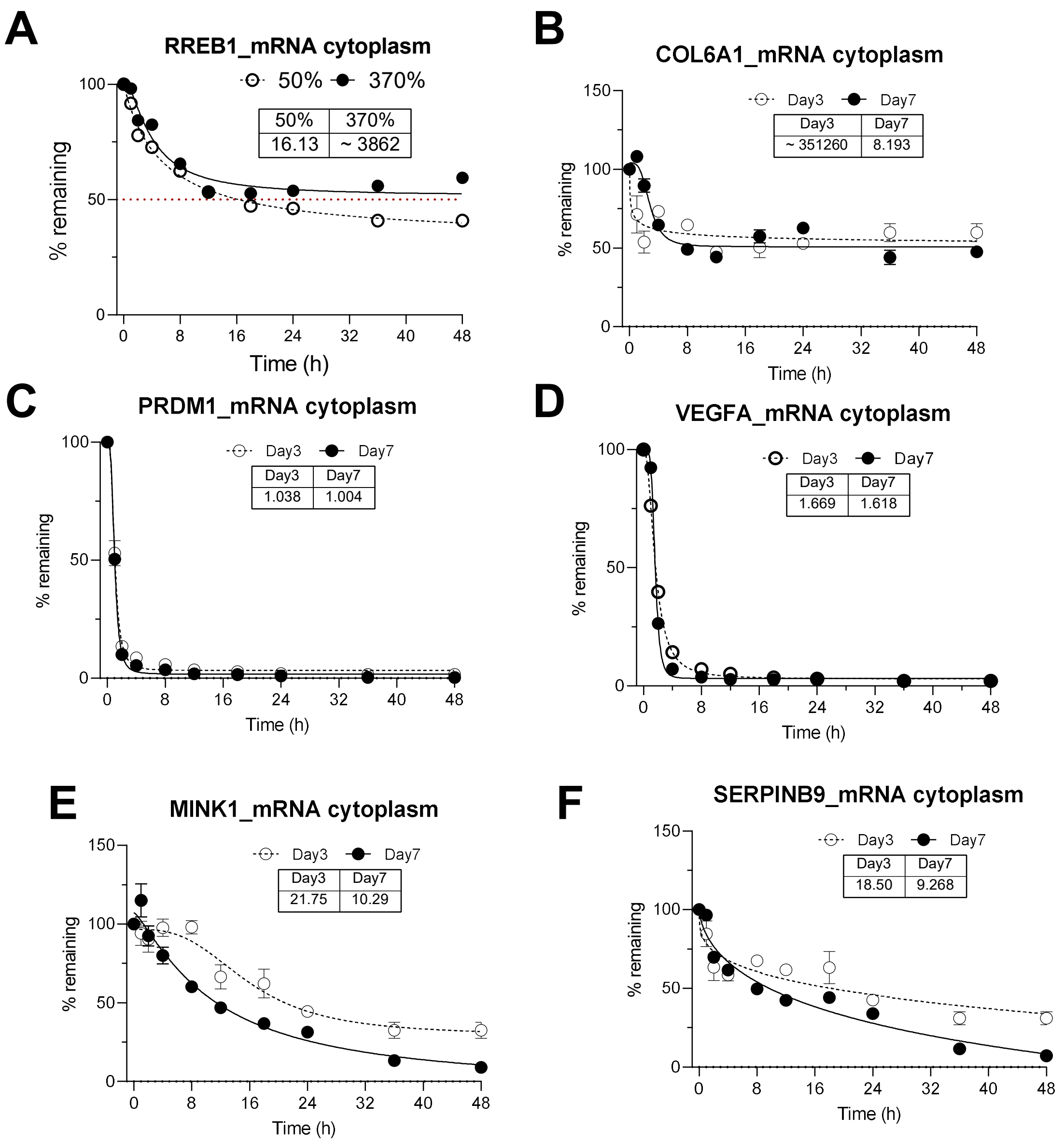

Supplemental Figure 13. (A-F) mRNA stability assays for upregulated targets in RNA isolated from cytoplasm fraction from cells harvested on Day 3 (50%) and Day 7 (370%). Table shows calculated estimate of half-life in hours on Day 3 and 7. (A-D) mRNAs had too long half-life or too short half-life to be compared or showed no difference between experimental conditions and (E-F) mRNAs showed longer half-life on Day 3 (50%).

NLS-AGO2 HCT116 construct

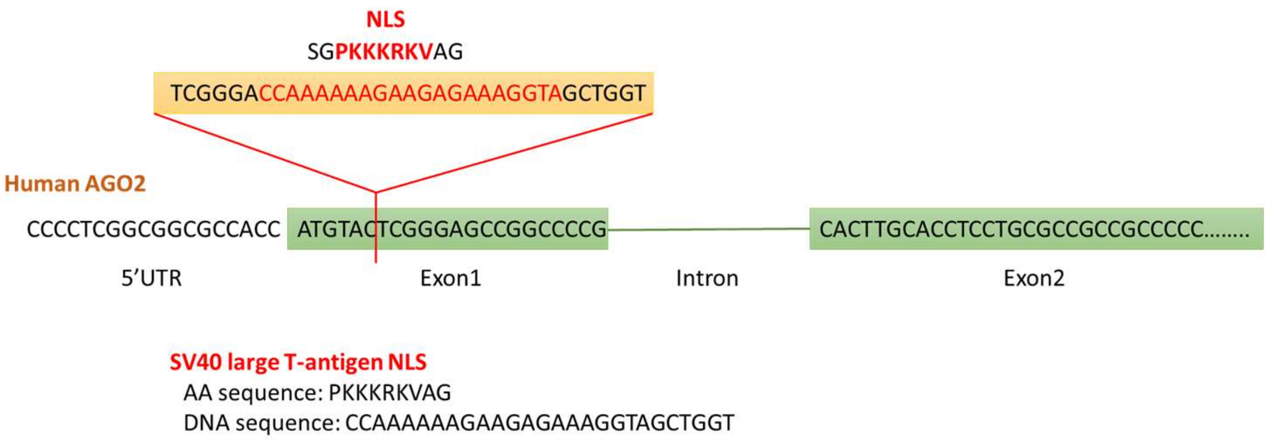

Supplemental Figure 14. CRISPR/Cas9-mediated NLS-AGO2 HCT116. SV40 NLS is inserted into N-terminal of human AGO2 according to previous papers. NLS fragment has 2 amino acid at both side as spacer sequences shown in black. “SG” on 5’ side of NLS and “AG” on 3’ side. NLS is inserted into both alleles to express AGO2 in only nucleus. To keep endogenous transcription level, endogenous promoter is used.

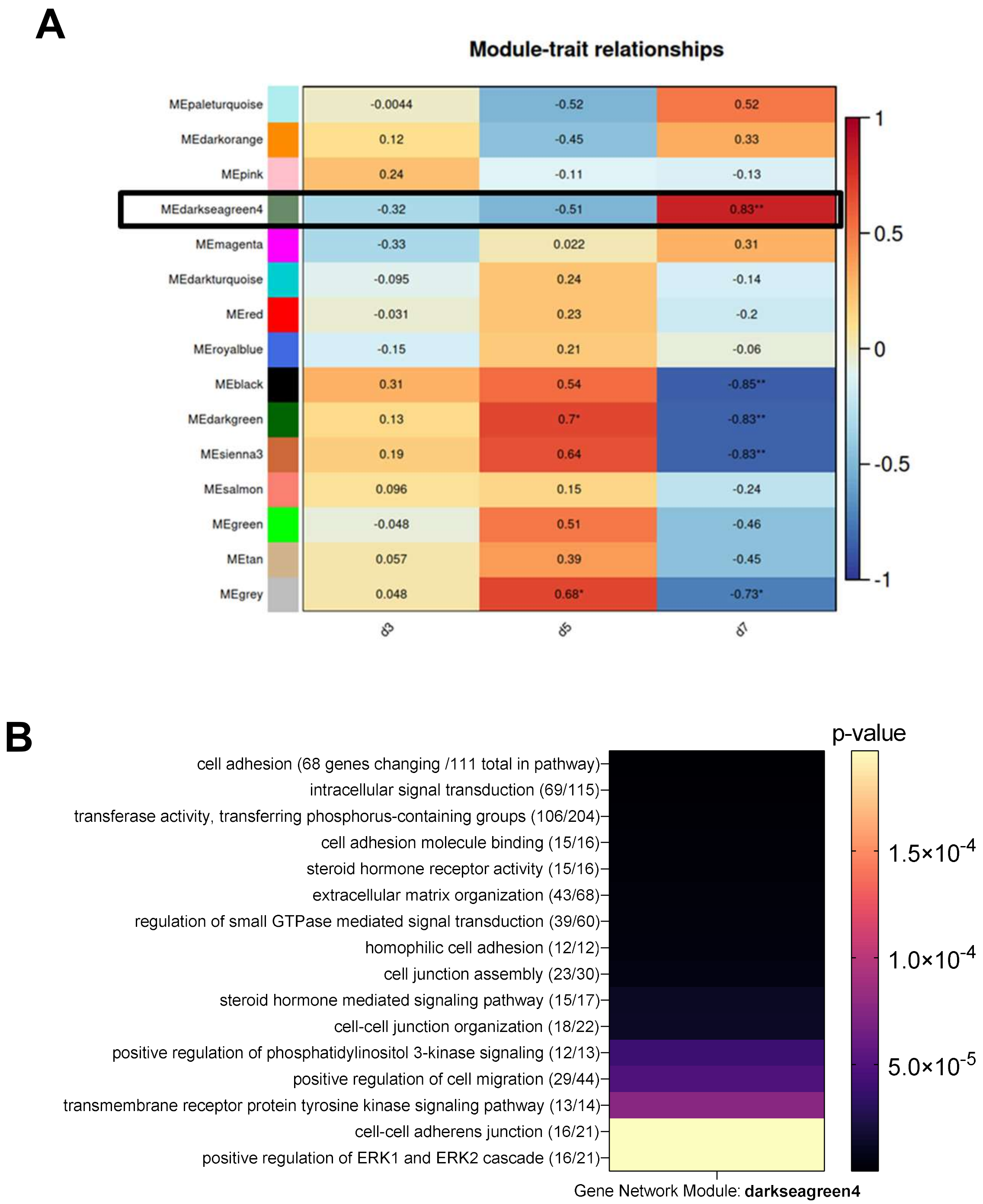

Supplementary Figure 15. (A) Results from Gene Network Analysis showing Pearson Correlation values for identifying groups/modules of genes named after different colors whose expression levels are positively or negatively correlated with different levels of cell density. D3 represents Day 3 at 50% confluency, D5 represents Day 5 at 250% confluency, and D7 represents Day 7 at 400% confluency. (B) Significance, p-value, of enriched pathways within "darkseagreen4" gene network module shown in black box in (A). Ratio in parentheses show number of genes with differential expression change out

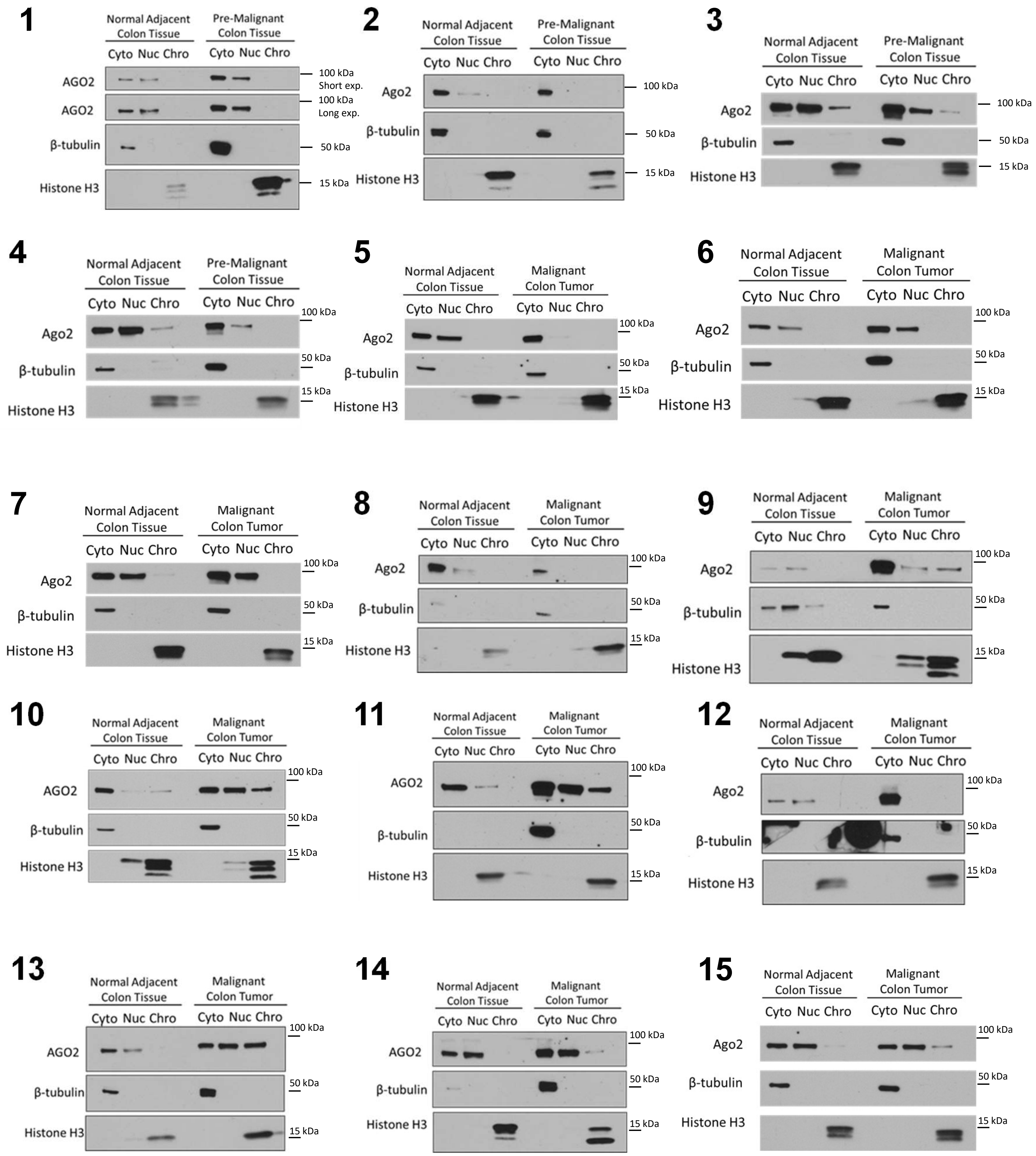
1
Normal Adjacent Colon Tissue
Pre-Malignant Colon Tissue
Cyto Nuc Chro Cyto Nuc Chro
AGO2
100 kDa Short exp.
AGO2
100 kDa Long exp.
β-tubulin
50 kDa
Histone H3
15 kDa
2
Normal Adjacent Colon Tissue
Pre-Malignant Colon Tissue
Cyto Nuc Chro Cyto Nuc Chro
Ago2
100 kDa
β-tubulin
50 kDa
Histone H3
15 kDa
3
Normal Adjacent Colon Tissue
Pre-Malignant Colon Tissue
Cyto Nuc Chro Cyto Nuc Chro
Ago2
100 kDa
β-tubulin
50 kDa
Histone H3
15 kDa
4
Normal Adjacent Colon Tissue
Pre-Malignant Colon Tissue
Cyto Nuc Chro Cyto Nuc Chro
Ago2
100 kDa
β-tubulin
50 kDa
Histone H3
15 kDa
5
Normal Adjacent Colon Tissue
Malignant Colon Tumor
Cyto Nuc Chro Cyto Nuc Chro
Ago2
100 kDa
β-tubulin
50 kDa
Histone H3
15 kDa
6
Normal Adjacent Colon Tissue
Malignant Colon Tumor
Cyto Nuc Chro Cyto Nuc Chro
Ago2
100 kDa
β-tubulin
50 kDa
Histone H3
15 kDa
7
Normal Adjacent Colon Tissue
Malignant Colon Tumor
Cyto Nuc Chro Cyto Nuc Chro
Ago2
100 kDa
β-tubulin
50 kDa
Histone H3
15 kDa
8
Normal Adjacent Colon Tissue
Malignant Colon Tumor
Cyto Nuc Chro Cyto Nuc Chro
Ago2
100 kDa
β-tubulin
50 kDa
Histone H3
15 kDa
9
Normal Adjacent Colon Tissue
Malignant Colon Tumor
Cyto Nuc Chro Cyto Nuc Chro
Ago2
100 kDa
β-tubulin
50 kDa
Histone H3
15 kDa
10
Normal Adjacent Colon Tissue
Malignant Colon Tumor
Cyto Nuc Chro Cyto Nuc Chro
AGO2
100 kDa
β-tubulin
50 kDa
Histone H3
15 kDa
11
Normal Adjacent Colon Tissue
Malignant Colon Tumor
Cyto Nuc Chro Cyto Nuc Chro
AGO2
100 kDa
β-tubulin
50 kDa
Histone H3
15 kDa
12
Normal Adjacent Colon Tissue
Malignant Colon Tumor
Cyto Nuc Chro Cyto Nuc Chro
Ago2
100 kDa
β-tubulin
50 kDa
Histone H3
15 kDa
13
Normal Adjacent Colon Tissue
Malignant Colon Tumor
Cyto Nuc Chro Cyto Nuc Chro
AGO2
100 kDa
β-tubulin
50 kDa
Histone H3
15 kDa
14
Normal Adjacent Colon Tissue
Malignant Colon Tumor
Cyto Nuc Chro Cyto Nuc Chro
AGO2
100 kDa
β-tubulin
50 kDa
Histone H3
15 kDa
15
Normal Adjacent Colon Tissue
Malignant Colon Tumor
Cyto Nuc Chro Cyto Nuc Chro
Ago2
100 kDa
β-tubulin
50 kDa
Histone H3
15 kDa

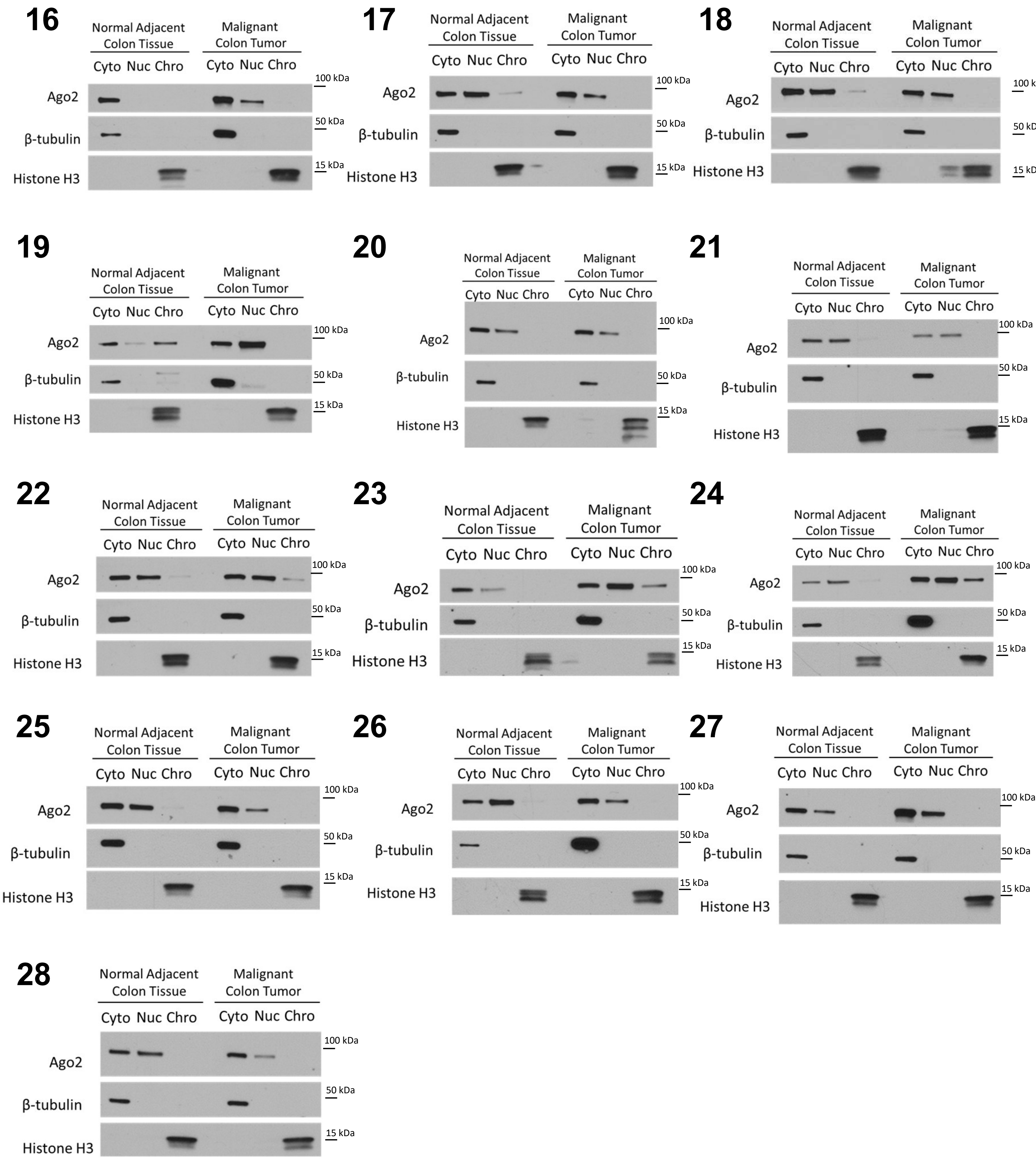

Supplemental Figure 16. AGO2 Localization in Human Colon Tissue.
(1-28) Western blot from pairs of tissue from Patients #1-28 fractionated into cytoplasm (cyto), soluble nuclear (nuc), and chromatin (chro) fractions. Pre-malignant indicates a colon adenoma tissue sample. Malignant indicates a colon adenocarcinoma tissue sample.

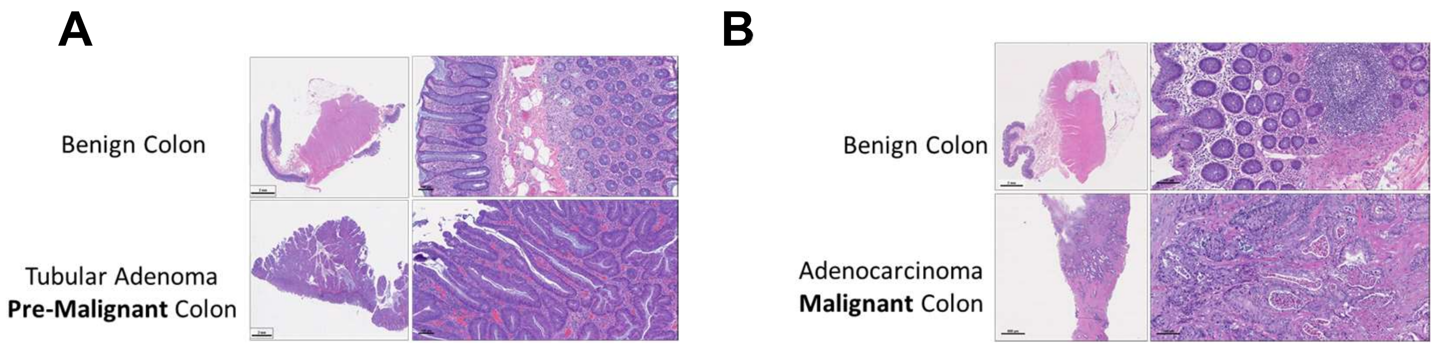

Supplemental Figure 17. (A, B) H&E staining of tissue slices from (A) Patient #2 and (B) Patient #10 used for pathology analysis.
